# Supplementary material for: Genetic mapping and identification of a QTL determining tolerance to freezing stress in Fragaria vesca L
Source: PLoS One. 2021 May 21;16(5):e0248089. doi: 10.1371/journal.pone.0248089 (PMC8139484; doi:10.1371/journal.pone.0248089)
Supplement: S4 Table — (DOCX) [file pone.0248089.s004.docx]

S4 Table. Selected gene features in the QTL region exhibiting transcript accumulation differences in crowns and leaves of genotypes ‘Alta’ and ‘NCGR1363.’

|  | **Gene ID** | **Description** | **FC 42 days (d) vs. 0 hours (h) LTS** | | | | **FD NCGR1363 vs. Alta @ 0 h LTS** | | **FD NCGR1363 vs. Alta @ 42 d LTS** | |
| --- | --- | --- | --- | --- | --- | --- | --- | --- | --- | --- |
|  |  |  | **Alta**  **crown** | **NCGR1363 crown** | **Alta**  **leaf** | **NCGR1363 leaf** | **Crown** | **Leaf** | **Crown** | **Leaf** |
| **A** | *FvH4_2g11510*^t3^ | dynamin-related protein 3A (DRP3A) | -1.2* | NA | -1.5* | NA | -2049.1^+^ | -46.1* | -1641.4 | -72.7 |
|  | *FvH4_2g12511* | hAT dimerization-like protein | -182.1 | -2.3 | NA | NA | 3.6 | NA | 301.4 | NA |
|  | *FvH4_2g13680* | BYPASS1-like | -7.4^+^ | NC* | 1.2* | -1.7 | -6.3^+^ | 1.7 | 1.2* | -1.3* |
|  | *FvH4_2g14750* | alcohol dehydrogenase-like | 3.0* | -1.3^+,t2^ | 4.4^+^ | 1.2* | 2.4^+^ | -1.3* | -1.6* | -4.9^+^ |
|  | *FvH4_2g14760* | alcohol dehydrogenase | 13.7* | 2.5^+,t1^ | 48.1^+^ | 15.9^+^ | 2.5^+^ | -1.8 | -2.2* | -5.7^+^ |
|  | *FvH4_2g14860* | sugar transporter SWEET1-like | 8.6^+^ | 2.8 | 3.2^+^ | 1.5* | -2.9^+^ | NC | -9.0^+^ | -2.2* |
|  | *FvH4_2g15440*^t3^ | aquaporin PIP2-7-like | -4.0* | -1.4* | 4.4 | 1.2* | -3.6^+^ | 2.1* | -2.8* | -1.7* |
|  | *FvH4_2g15800* | ser/thr prot. kinase CTR1 | NC* | NC* | 3.3^+^ | 1.1* | -5.2^+^ | 1.3* | -5.5* | -2.5* |
|  | *FvH4_2g15840* | N-acetylserotonin O-methyltransferase (ASMT) | -1195.1^+^ | -11.2 | NA | NA | -2.5^+^ | NA | NA | NA |
|  | *FvH4_2g16000* | L-ascorbate oxidase homolog | -2.0* | -1.1* | -5.7^+^ | -1.8* | -11.7 | -2.2* | -6.5* | 1.4* |
|  | *FvH4_2g16110* | expansin-like A2 | -2.9* | 5.7^+^ | 7.2^+^ | 4.5^+^ | -49.6^+^ | -3.1^+^ | -3.1* | -5.3^+^ |
|  | *FvH4_2g16170* | E3 ubiquitin ligase RDUF2 | 1.2* | NA | -1.1* | NA | -220.2^+^ | -667.9^+^ | -865.5 | -704.1^+^ |
|  | *FvH4_2g16180* | transcription factor NAC029 | -8.2^+^ | -2.9^+^ | -1.8* | -3.7 | -4.1^+^ | 2.1* | -1.5* | ND* |
|  | *FvH4_2g18440* | ninja-family protein AFP2-like | 11.9* | 1.5* | 124.8^+^ | 11.1^+^ | ND* | NA | -8.0* | -5.1 |
| **B** | *FvH4_2g11650* | transcription factor ORG2-like | -8.1* | -26.8^+^ | -46.6^+^ | NA | -1.1* | -1.3* | NA | NA |
|  | *FvH4_2g12040* | splicing factor 3A subunit 3-like | 1.3* | 1.3* | 1.3* | 1.7* | -6.6 | -5.2 | -6.4 | -4.2 |
|  | *FvH4_2g12161* | SAM-dependent methyltransferase | 1.6* | -1.5 | -1.1* | -1.8 | -1.6 | 1.1* | -3.9 | -1.7* |
|  | *FvH4_2g12540* | ser/thr prot.-kinase LRK10L1.2 | -1.3* | 1.2 | 1.5 | NC* | 1.8 | 5.3 | 2.9 | 3.2 |
|  | *FvH4_2g12570* | putative laccase 9 | -2.6* | 1.1* | 12.4^+^ | 6.5* | -21.0^+^ | NA | -7.1* | -1.8* |
|  | *FvH4_2g12620* | laccase-14-like | 73.1* | 1.1* | -18.0^+^ | -1.4* | 16.1^+^ | ND* | -4.3* | 12.1* |
|  | *FvH4_2g13240* | transcription factor ABR1-like | 1.7* | 2.1* | 312.5^+^ | 51.7 | 30.9^+^ | NA | -24.1* | -9.9* |
|  | *FvH4_2g13960* | vacuolar iron transporter homolog 2-like | -21.3 | -2.3 | -5.8 | NC* | 1.1* | -1.9* | 10.4* | 2.9* |
|  | *FvH4_2g14190* | carbonic anhydrase 2-like | 33.9* | NA | -79.2^+^ | -21.0^+^ | -43.7^+^ | 5.1* | NA | 18.4 |
|  | *FvH4_2g14690* | acyl-[acyl-carrier-protein] | -181.8 | -28.8^+^ | NA | NA | 9.3^+^ | NA | 57.9* | NA |
|  | *FvH4_2g14900* | thioredoxin M-type | 82.1* | 5.8^+^ | -2.7^+^ | -1.3* | -2.3^+^ | ND* | -31.9* | 1.9* |
|  | *FvH4_2g15730* | Major facilitator superfamily | 6.2 | 1.3 | 1.6 | -1.1* | 2.3 | 1.8 | -2.1* | ND* |
|  | *FvH4_2g16430* | flavin-containing monooxygenase 1 | 61.0 | 8.9^+^ | 1.5 | 8.2^+^ | NA | -8.0^+^ | -7.3* | -1.6* |
|  | *FvH4_2g16500* | cytochrome P450 87A3-like | -2.4* | -1.7 | NA | -1.3* | 27.6^+^ | 11132.5^+^ | 39.3* | 9688.2 |
|  | *FvH4_2g16690* | bromodomain-containing protein 9-like | NA | NA | 3.71^+^ | 1.6^+^ | -1.4^+^ | 1.5^+^ | -1.4* | -1.6* |
|  | *FvH4_2g17051* | MAD3 / HMGR1 | -10.6^+^ | -1.1* | -1.2* | -1.1* | -7.6^+^ | -1.1* | 1.3* | -1.1* |
|  | *FvH4_2g17060* | MAD3 / HMGR1 | -86.3^+^ | -1.7 | -1.6* | -3.1* | -64.5^+^ | 1.5* | -1.2* | -1.4* |
|  | *FvH4_2g18040* | Ser/thr prot. kinase OXI1 | -2.7* | -1.2* | -2.5^+^ | -2.0 | -8.4^+^ | -1.3* | -2.6* | -1.1* |
|  | *FvH4_2g18210* | lyzozyme D-like | -2.2* | 2.8^+^ | -2.7^+^ | -36.5^+^ | -24.9^+^ | -1.5* | -4.0* | -21.6 |

Gene features with known (A) and less-known (B) roles in plant response to low temperature stress (LTS); FC, fold change; FD, fold difference; ^+^FC & FD values are significant at FDR-adjusted p-values ≤ .01, all others at ≤ .05 except where

denoted (*, i.e. > .05) and all reflect gene level expression except mRNA isoform levels for ^t3^*FvH4_2g11510.t3, FvH4_2g15440.t3,* ^t2^*FvH4_2g14750.t2 and* ^t1^*FvH4_2g14760.t1*; NA, not available (detected); NC, no change; ND, no difference
